# A Systematic Review and Meta-Analysis of the Prevalence of Triplex Infections (Combined Human Immunodeficiency Virus, Hepatitis B Virus, and Hepatitis C Virus) among Pregnant Women in Nigeria

**DOI:** 10.1155/2023/3551297

**Published:** 2023-07-17

**Authors:** George Uchenna Eleje, Olabisi Morebise Loto, Hadiza Abdullahi Usman, Chinyere Ukamaka Onubogu, Preye Owen Fiebai, Godwin Otuodichinma Akaba, Ayyuba Rabiu, Ikechukwu Innocent Mbachu, Moriam Taiwo Chibuzor, Rebecca Chinyelu Chukwuanukwu, Ngozi Nneka Joe-Ikechebelu, Emeka Philip Igbodike, Richard Obinwanne Egeonu, Ijeoma Chioma Oppah, Uchenna Chukwunonso Ogwaluonye, Chike Henry Nwankwo, Stephen Okoroafor Kalu, Chisom God'swill Chigbo, Chukwuanugo Nkemakonam Ogbuagu, Shirley Nneka Chukwurah, Chinwe Elizabeth Uzochukwu, Aishat Ahmed, Chiamaka Henrietta Jibuaku, Samuel Oluwagbenga Inuyomi, Bukola Abimbola Adesoji, Ubong Inyang Anyang, Ekene Agatha Emeka, Odion Emmanuel Igue, Ogbonna Dennis Okoro, Prince Ogbonnia Aja, Chiamaka Perpetua Chidozie, Hadiza Sani Ibrahim, Fatima Ele Aliyu, Harrison Chiro Ugwuoroko, Aisha Ismaila Numan, Solace Amechi Omoruyi, Osita Samuel Umeononihu, Chukwuemeka Chukwubuikem Okoro, Ifeanyi Kingsley Nwaeju, Arinze Anthony Onwuegbuna, Lydia Ijeoma Eleje, David Chibuike Ikwuka, Eric Okechukwu Umeh, Sussan Ifeyinwa Nweje, Ifeoma Clara Ajuba, Angela Ogechukwu Ugwu, Uzoamaka Rufina Ebubedike, Divinefavour Echezona Malachy, Chigozie Geoffrey Okafor, Nnaedozie Paul Obiegbu, Emmanuel Onyebuchi Ugwu, Ibrahim Adamu Yakasai, Oliver Chukwujekwu Ezechi, Joseph Ifeanyichukwu Ikechebelu

**Affiliations:** ^1^Department of Obstetrics and Gynecology, Nnamdi Azikiwe University, Awka, Nigeria; ^2^Department of Obstetrics and Gynecology, Nnamdi Azikiwe University Teaching Hospital, PMB 5025, Nnewi, Anambra State, Nigeria; ^3^Department of Obstetrics and Gynecology, Obafemi Awolowo University, Ile-Ife, Nigeria; ^4^Department of Obstetrics and Gynecology, Obafemi Awolowo University Teaching Hospital Complex, Ile-Ife, Nigeria; ^5^Department of Obstetrics and Gynecology, University of Maiduguri, Maiduguri, Nigeria; ^6^Department of Obstetrics and Gynecology, University of Maiduguri Teaching Hospital, Maiduguri, Nigeria; ^7^Department of Paediatrics, Nnamdi Azikiwe University, Awka, Nigeria; ^8^Department of Obstetrics and Gynecology, University of Port Harcourt, Port Harcourt, Nigeria; ^9^Department of Obstetrics and Gynecology, University of Port Harcourt Teaching Hospital, Port Harcourt, Nigeria; ^10^Department of Obstetrics and Gynecology, University of Abuja, Abuja, Nigeria; ^11^Department of Obstetrics and Gynecology, University of Abuja Teaching Hospital, Abuja, Nigeria; ^12^Department of Obstetrics and Gynecology, Bayero University, Kano, Nigeria; ^13^Department of Obstetrics and Gynecology, Aminu Kano Teaching Hospital, Kano, Nigeria; ^14^Cochrane Nigeria, Institute of Tropical Diseases Research and Prevention, University of Calabar Teaching Hospital, Calabar, Nigeria; ^15^Immunology Unit, Department of Medical Laboratory Science, Nnamdi Azikiwe University, Awka, Nigeria; ^16^Department of Community Medicine and Primary Health Care, Faculty of Medicine, Chukwuemeka Odumegwu Ojukwu University, Amaku, Awka, Nigeria; ^17^Department of Community Medicine and Primary Health Care, Faculty of Medicine, Chukwuemeka Odumegwu Ojukwu University Teaching Hospital, Amaku, Awka, Nigeria; ^18^Department of Obstetrics and Gynecology, Havana Specialist Hospital, Surulere Lagos, Nigeria; ^19^Department of Pharmaceutical Sciences, Nnamdi Azikiwe University, Awka, Nigeria; ^20^Department of Statistics, Nnamdi Azikiwe University, Awka, Nigeria; ^21^HIV Care Laboratory, HIV Care Department, Nnamdi Azikiwe University Teaching Hospital, Nnewi, Nigeria; ^22^School of Public Health, University of Port Harcourt, Port Harcourt, Rivers State, Nigeria; ^23^Department of Medical Microbiology and Parasitology, Faculty of Medicine, Nnamdi Azikiwe University, Awka, Nigeria; ^24^Gastroenterology Unit, Department of Medicine, Faculty of Medicine, Nnamdi Azikiwe University, Awka, Nigeria; ^25^Department of Mass Communication, Nnamdi Azikiwe University, Awka, Nigeria; ^26^Department of Physics and Engineering Physics, Obafemi Awolowo University, Ile-Ife, Nigeria; ^27^Department of Nursing, Obafemi Awolowo University Teaching Hospital Complex, Ile-Ife, Nigeria; ^28^Department of Family Medicine, Faculty of Medicine, Nnamdi Azikiwe University, Awka, Nigeria; ^29^Department of Physiological Sciences, Obafemi Awolowo University, Ile-Ife, Nigeria; ^30^Department of Parasitology & Entomology, Faculty of Veterinary Medicine, University of Maiduguri Borno State, Maiduguri, Nigeria; ^31^Department of Ophthalmology, Nnamdi Azikiwe University, Awka, Nigeria; ^32^Measurement Evaluation and Research Unit, Department of Educational Foundations, Nnamdi Azikiwe University, Awka, Nigeria; ^33^Department of Human Physiology, Nnamdi Azikiwe University, Awka, Nigeria; ^34^Department of Radiology, Faculty of Medicine, Nnamdi Azikiwe University, Awka, Nigeria; ^35^Department of Nursing, Nnamdi Azikiwe University Teaching Hospital, Nnewi, Nigeria; ^36^Department of Hematology, Faculty of Medicine, Nnamdi Azikiwe University, Awka, Nigeria; ^37^Department of Hematology & Immunology, College of Medicine, University of Nigeria, Ituku-Ozalla, Enugu State, Nigeria; ^38^Department of Obstetrics and Gynecology, College of Medicine, University of Nigeria Enugu Campus, Enugu, Nigeria; ^39^Nigerian Institute of Medical Research, Lagos, Nigeria

## Abstract

**Objective:**

We systematically identified the prevalence of triplex infections (combined human immunodeficiency virus (HIV), hepatitis B virus (HBV), and hepatitis C virus (HCV)) in pregnancy.

**Methods:**

To gather information on the frequency of triplex infections, we searched the databases of PubMed, CINAHL, and Google Scholar. Without regard to language, we utilized search terms that covered HIV, HBV, HCV, and pregnancy. Pregnant women with triplex infections of HIV, HBV, and HCV were included in studies that also examined the prevalence of triplex infections. Review Manager 5.4.1 was employed to conduct the meta-analysis. Critical appraisal and bias tool risk data were provided as percentages with 95% confidence intervals (95% CIs), and *I*^2^ was used as the statistical measure of heterogeneity. The checklist was created by Hoy and colleagues. The study protocol was registered on PROSPERO, under the registration number CRD42020202583.

**Results:**

Eight studies involving 5314 women were included. We identified one ongoing study. Pooled prevalence of triplex infections was 0.03% (95% CI: 0.02–0.04%) according to meta-analysis. Subgroup analysis demonstrated a significantly high prevalence of 0.08% (95% CI: 0.06–0.10%; 3863 women) in HIV-positive population than 0.00% (95% CI:−0.00-0.00; 1451 women; *P* < 0.001) in general obstetric population. Moreover, there was a significant difference in the pooled prevalence between studies published between 2001 and 2010 and between 2011 and 2021 (0.14% (95% CI: 0.12 to 0.16 versus 0.03% (95% CI: 0.02 to 0.04%; *P* < 0.001))) and participants recruited in the period between 2001 and 2011 and between 2012 and 2021 (0.13% (95% CI: 0.05 to 0.21; *p*=0.002 versus 0.00% (95% CI: −0.00 to 0.00%; *p*=1.00))), respectively.

**Conclusion:**

The combined prevalence of prenatal triplex infections was 0.03%, with rates notably higher among the group of pregnant women who were HIV-positive and during the recruitment period that took place before 2012. This prevalence still necessitates screening for these infections as necessary.

## 1. Introduction

Human immunodeficiency virus (HIV), hepatitis B virus (HBV), and hepatitis C virus (HCV) triplex infections in pregnant women continue to be serious public health issues [1, 2]. This is due to the serious health risks that triplex infection in pregnant women brings to both the mothers and the newborn babies [3]. It has continued to be a widespread, uncontrolled health problem and could be brought on by interactions between the immune system and the virus [4]. For instance, when HBV and HCV are coinfected, the health of pregnant women with HIV/acquired immune deficiency syndrome (AIDS) may rapidly deteriorate [5]. When compared to individuals with HIV-HBV only, HIV-HCV only, or mono HIV infection, patients who were triply infected with HIV/HBV/HCV seemed to have lower CD4+ levels [6].

Although HIV and AIDS were once thought to be terminal illnesses, highly active antiretroviral therapy (HAART) has transformed these conditions into a manageable chronic infection by lowering the rates of mother-to-child (MTCT) HIV transmission. Since hepatitis viruses are known to have adverse effects on people with HIV, triplex infections may negate the positive effects of HAART [6]. As a result, hepatitis virus and HIV double or triple infections require more attention, especially in low- and middle-income nations like Nigeria where these virus combinations are common [6].

Although there may be fewer pregnant women who have triplex infections, the combination of HIV, HBV, and HCV is an unacceptable coexistence [7] and could have a negative impact on MTCT rates [8]. Pregnant women's triplex infection with HIV, HBV, and HCV is thus a cause for concern [9, 10]. One of the nations with a high prevalence of HIV and viral hepatitis is Nigeria [11]. By defining goals for achieving significant reductions in new infections and mortality from HIV and viral hepatitis by 2030, the World Health Organization (WHO) has patented the worldwide get-up-and-go of eliminating viral hepatitis infection. Therefore, the WHO recommended triple elimination in pregnant women and the elimination of viral hepatitis by 2030, while hepatitis C was not included. The attainment of WHO targets, particularly in sub-Saharan Africa, has suffered significant failures in this global endeavor to eradicate HIV and viral hepatitis [10]. In addition, the global triplex infection pandemic caused by the coronavirus disease 2019 (COVID-19) pandemic has affected healthcare systems and access to care [12]. In 32% (32/101) of the countries, access to HIV care and antiretroviral medication has been hampered [12]. These shortcomings and difficulties have been noted by experts in HIV and viral hepatitis, who have also emphasized the need for epidemiological studies to follow evidence-based research practices [9, 12].

To the best of the authors' knowledge, there has not yet been published data on the prevalence of triplex infections (combined HIV, HBV, and HCV infections) among expectant women. Thus, the purpose of the study is to estimate the pooled prevalence of triplex HIV, HBV, and HCV infections among pregnant women.

## 2. Methods

### 2.1. Study Protocol and Registration

The study protocol was registered on PROSPERO, under the registration number CRD42020202583.

### 2.2. Study Design

In the current study, there has been a thorough review and meta-analysis. The revised Preferred Reporting Items for Systematic Reviews and Meta-Analyses (PRISMA-P) standards were followed in the reporting of this review.

### 2.3. Setting

Nigeria is a nation in West Africa. It is divided into five geopolitical regions: the north-central area, where the capital city Abuja is located, the south-east, south-south, south-west, and north-east. In 2018, there were more than 200 million people living in Nigeria.

### 2.4. Study Population

The study population was defined as pregnant women with triplex infections of HIV, hepatitis B, and hepatitis C viruses.

## 3. Selection Criteria

### 3.1. Criteria for considering Studies for the Review

#### 3.1.1. Inclusion Criteria

*(1) Study Design*. Cross-sectional, case-control, or cohort studies.

*(2) Participants*. Pregnant women with triplex infections of HIV, hepatitis B, and hepatitis C viruses residing in Nigeria.

*(3) Studies of Interest*. Studies reporting the seroprevalence of HIV-HBV-HCV triplex infections (anti-HIV antibodies I and II, HBsAg and/or infectivity (HBe antigen (HBeAg)), and/or HCV infection markers (HCVAb and/or HCV detectable viral load) in pregnancy or enough data to compute this estimate in pregnancy even though the prevalence data are missing, i.e., if the prevalence data are missing but there is sufficient data to calculate the prevalence, the study was included.

*(4) Outcome Measurement*. Results were measured by looking at the presence of anti-HIV antibodies I and II, HBsAg, and/or infectivity (HBe antigen), as well as anti-HCV.

*(5) Types of Publication*. Published and unpublished data.

#### 3.1.2. Exclusion Criteria

Studies carried out among pregnant Nigerian women, populations living outside of Nigeria, and nonpregnant populations were all eliminated. Studies in subgroups of participants chosen based on the presence of any other viral hepatitis other than HIV-HBV-HCV triplex infection, such as case series (20 participants) of the hepatitis D (delta) virus (HDV), were also excluded. Studies reporting only mono-infection (only HIV, HBV, or HCV), or only dual infections in pregnancy, were also excluded, as were reviews, letters, commentaries, and editorials. In addition, studies lacking primary data and/or explicit method description after two unsuccessful requests addressed to the corresponding author were also excluded. The study was also disqualified if the abstract lacked conclusion and the full text was unavailable. The most thorough and recent version was utilized for duplicate publications (research published in more than one report).

#### 3.1.3. Search Strategy

The new PRISMA-P (Preferred Reporting Items for Systematic Reviews and Meta-Analysis Protocols) standard was used when conducting this review [13]. A thorough search of the PubMed, Google Scholar, and CINAHL databases was conducted to find all pertinent papers on HIV, HBV, and HCV dual and triple infections in pregnancy published from 1 February 2001 to 31 January 2021 without regard to language. See Appendix S1 for the complete search plan. If studies were not reported in English (the reviewers' native tongue), we looked for translations. The studies were listed as being awaiting classification if translations could not be available. To get the data that were current, we restricted the search to the previous 20 years. The search terms included synonyms and controlled vocabulary for “human immunodeficiency virus,” “hepatitis C,” “hepatitis B,” triplex infection^*∗*^, triple infection, pregnant women, and Nigeria. For the full search strategy, see Appendix S1.

#### 3.1.4. Searching Other Sources

Reference lists of eligible articles and relevant reviews were manually searched to identify additional studies.

## 4. Main Outcomes

### 4.1. Primary

The pooled prevalence rates of HIV-HBV-HCV triplex infection among pregnant women in Nigeria are the primary outcome of the study.

### 4.2. Analysis of Subgroups or Subsets

For research comparing participants from the HIV-positive group to the general prenatal population, studies from 2001 to 2010 to studies from 2011 to 2021, and studies from 2001 to 2011 to studies from 2012 to 2021, subgroup analyses were conducted. Only pregnant women with HIV-positive status were recruited or studied in the HIV-positive study population by the authors of the primary studies. However, in the general obstetric population (general obstetric study population), the authors of the primary research recruited or studied pregnant women without taking into account whether they are HIV-positive or not.

### 4.3. Selection of Included Studies

The software EndNote version X9 was used to import the retrieved articles and check them for duplication. Two researchers (GE and OL) independently reviewed each record's titles and abstracts to determine whether they should be included in the review. Two researchers (HU and GA) acquired the complete texts of papers deemed potentially eligible and evaluated them for eligibility. Two investigators mutually agreed to keep studies for inclusion. Any differences of opinion were settled by a third researcher (PF).

### 4.4. Data Extraction

A pretested data extraction form created in Microsoft Excel was used by two independent reviewers (CO and RE), with a third reviewer (IM) resolving any differences. First author, year of publication, period of participant recruitment, region of recruitment, site, setting, timing of data collection, study design, eligibility requirements, sample size, mean or median age, diagnostic criteria, number of samples tested for HIV, HCV, and/or HBV, number of participants with HIV I and II, HBsAg, HBeAg, HCVAb, and HCV detectable viral load, and prevalence rate were among the pertinent information that the reviewers independently extracted.

HIV I and II, HBsAg, or HCVAb for HIV, HBV, and HCV, respectively, were used to define infection. HIV I and II for HIV, HBsAg, and/or HBeAg for HBV, and both HCVAb and detectable viral load for HCV were considered indicators of infection. On the other hand, in order to estimate the percentage of pregnant women who can pass the virus onto their unborn child, we reported the prevalence of HIV I and II, HBeAg, and HCV detectable viral load, respectively, among HIV, HBV, and HCV positive women. We made at least two separate attempts to get in touch with the study's corresponding author personally when pertinent data were not readily available.

### 4.5. Methodological Quality and Risk of Bias Assessment

Using a modified version of the risk of bias instrument for prevalence studies, which was created by Hoy and colleagues [14], the methodological quality and bias risk of the included studies were evaluated by two independent reviewers (EI and IO, with disagreements handled by a third reviewer (UO)). The study's target population had to be described in detail, as well as the sampling frame, sampling methods, response rate, nonproxy data collection, case definition, validity and reliability of the study instrument, mode of data collection, and an appropriate description of the numerator and denominator for the parameter of interest. These nine quality domains were also included.

Each chosen study received a special identification. The overall score was classed as follows, with a total score ranging from 0 to 9: 0–3: “low risk,” 4–6: “moderate risk,” and 7–9: “high risk” of bias. Better quality was indicated by a lower score.

### 4.6. Data Synthesis and Analysis

The Nordic Cochrane Center, Copenhagen, Denmark, provided Review Manager 5.4.1 software for use in all statistical calculations. For the entire study as well as for the various subgroups, the findings were presented as pooled prevalence with 95% confidence intervals (CI). The frequency of HIV, HBV, and HCV positive laboratory results was used to determine prevalence. The prevalence of triplex infection in the obstetric population was pooled, and the percentage (with a 95% confidence interval) was utilized as the effect size. The pooled effect was then calculated using the generic inverse variance approach, because the effect of a single rate and its standard error are similar to the rate difference (RD) at this time [15]. The Q test and forest plots were used to evaluate the degree of study heterogeneity. We utilized the fixed effects model and believed that the included studies had good homogeneity if the *p* value for the heterogeneity test was >0.10. The random effects model was applied in the other cases. We conducted subgroup and meta-regression analyses to look into potential sources of heterogeneity when significant heterogeneity (*I*^2^ > 75%) was found. *P* values less than 0.05 were regarded as statistically significant for all other tests other than the heterogeneity test.

## 5. Results

### 5.1. Description of Studies

**Figure 1:**
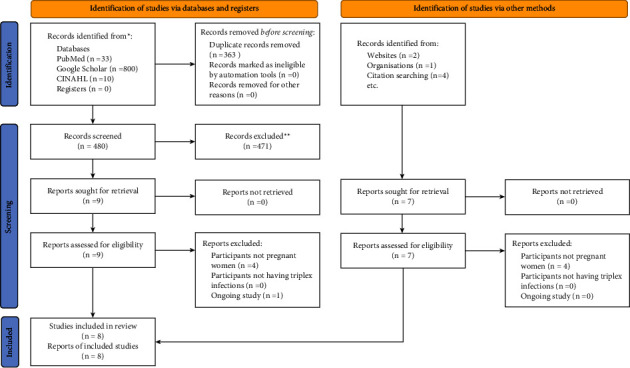
PRISMA flowchart. ^*∗*^Consider, if feasible to do so, reporting the number of records identified from each database or register searched (rather than the total number across all databases/registers). ^*∗∗*^If automation tools were used, indicate how many records were excluded by a human and how many were excluded by automation tools.

Additional hand searches also produced 7 citations, bringing the total number of results from the search approach to 843. Sixteen citations were chosen by the reviewers for full-text analysis after titles and abstracts were scrutinized. According to our selection criteria [9, 16–23], nine research were qualified (see Figure 1). Ultimately, eight of nine compatible citations [16–23] were included (Table 1). One of the nine qualifying studies was an ongoing one, thus it was excluded [9]. In Table 2 [5, 6, 24–28], the remaining seven excluded studies are listed together with the justifications for their exclusion. Participants in the included studies were enlisted between January 2006 and December 2018.

The eight included studies' [16–23] primary characteristics are also shown in Table 1. Only one study, 1/8 (12.5%), was a longitudinal cohort study, while the majority of the studies were cross-sectional 4/8 (50.0%) [17, 21–23] or retrospective cross-sectional studies 3/8 (37.5%) [16, 18, 20]. One study (1/8; 12.5%) was published between 2001 and 2010 [16], while the majority of the studies in our meta-analysis (7/8; 87.5%) were published between 2011and 2021 [18–23].

In addition, the majority of studies (five out of eight; 62.5%) involved pregnant women in the HIV-positive obstetric population [16, 17, 19–21], while three studies (three out of eight; 37.5%) used pregnant women from the general obstetrics population to survey for the prevalence of HIV-HBV-HCV triplex coinfection among ANC attendees [18, 22, 23].

Table 3 lists the diagnostic procedures, eligibility requirements, and bias risk/quality of the studies that were included. Finally, 3/8 (37.5%) of the included studies used ELISA to detect HBsAg [16, 18, 20], 2/8 (25.0%) used RDT [19, 23], 1/8 (12.5%) used immunochromatographic technique [21], 1/8 (12.5%) study used both ELISA and immunochromatographic technique [22], and 1/8 (12.5%) study was nonspecific regarding the type of laboratory method [17]. Only one study [20] used the PCR Technique to confirm the diagnosis of HCV, and only one study [19] did not specify the method used to test for HIV.

### 5.2. Meta-Analysis Results

**Figure 2:**
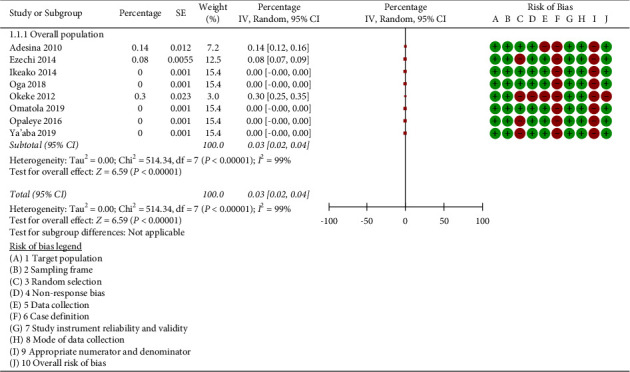
Meta-analysis showing the overall prevalence of triplex infection in pregnancy in the included studies.

When the heterogeneity of the eight studies was tested using Review Manager 5.4.1 software, *I*^2^ = 95%, *p* < 0.001 showed that there was significant heterogeneity among the studies. The random effects model was used as a result. According to the findings of our meta-analysis, the combined prevalence of triplex infection in the eight studies that were included was 0.03% (95% CI: 0.02–0.04%, *p* < 0.001; *I*^2^ = 99.0%) (Figure 2).

### 5.3. Sensitivity Analyses

**Figure 3:**
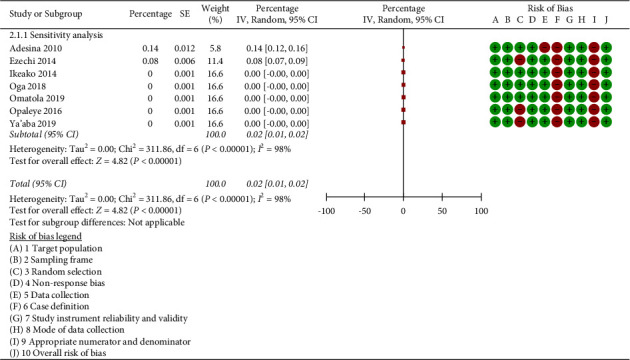
Meta-analysis showing the sensitivity analysis with prevalence of triplex infection in pregnancy with exclusion of study with high risk of bias.

Sensitivity analyses were used to evaluate the effect of specific studies on the aggregate prevalence. We omitted the studies with a moderate or high risk of bias because the research included in this review lacked sufficient uniformity. The pooled prevalence of triplex infections in pregnancy reduced to 0.02% (95% CI: 0.01 to 0.02%; *p* < 0.001; *I*^2^ = 98.0%) when we eliminated one study at a time, but it remained the same overall (Figure 3).

### 5.4. Subgroup Analyses

**Figure 4:**
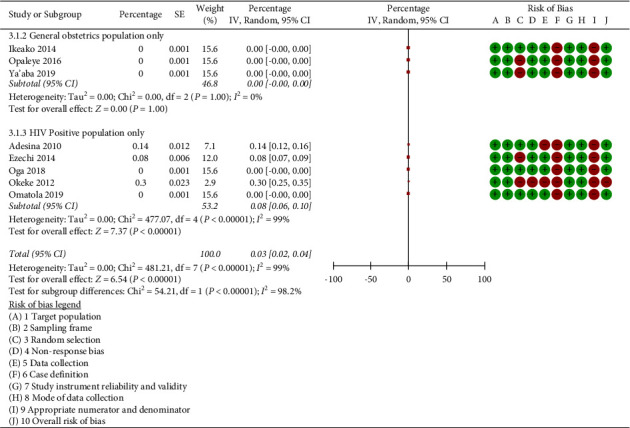
Subgroup analysis according to type of obstetric population.

Considering pregnant women who are HIV-positive may have different prevalence rates to the general obstetric population, we performed subgroup analyses for HIV-positive population and for the other general obstetric populations. The subgroup analyses revealed that the prevalence of triplex infection in the general obstetric population and the HIV-positive population were 0.0% (95% CI: −0.00-0.00; *I*^2^ = 0.0%) and 0.03% (95% CI: 0.02 to 0.04%, *p* < 0.001; *I*^2^ = 99.0%) respectively (Figure 4). Tests for subgroup differences showed significant difference (*p* < 0.001, *I*^2^ = 98.2%). The subgroup analysis did increase the heterogeneity, indicating that the HIV-positive study population is the source of heterogeneity (*I*^2^ = 0.0% vs 99.0%).

**Figure 5:**
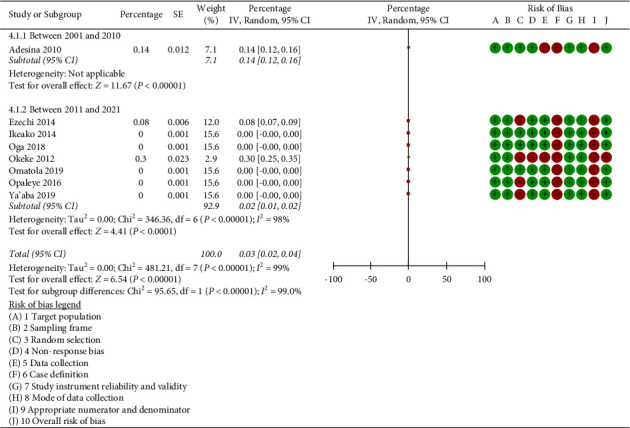
Subgroup analysis according to year of publication of the included studies.

A subgroup analysis of studies published between 2001 and 2010 and those published between 2011 and 2021 revealed that the prevalence of triplex infection was, respectively, 0.14% (95% CI: 0.12 to 0.16, *p* < 0.001) and 0.02% (95% CI: 0.01 to 0.02%, *p* < 0.001, *I*^2^ = 98.0%) in the studies published between 2001 and 2010 and in the studies published between 2011 and 2021 (see Figure 5). Significant differences were found in tests for subgroup differences (*p* < 0.001, *I*^2^ = 99%). The fact that the heterogeneity was not reduced by the subgroup analysis (*I*^2^ = 98.0% vs. 99.0%) shows that the year of publication is not the cause of the heterogeneity.

**Figure 6:**
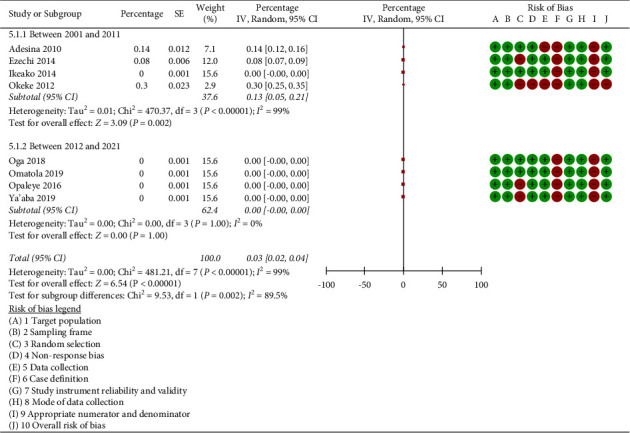
Subgroup analysis according to year of recruitment of participants in the included studies.

Figure 6 distinguishes itself by displaying the subgroup analysis in accordance with the year when participants in the included studies were recruited. It showed that the prevalence of triplex infection was 0.13% (95% CI: 0.05 to 0.21; *p*=0.002*; I*^2^ = 99.0%) and 0.00% (95% CI: −0.00 to 0.00%; *p*=1.00*; I*^2^ = 0.0%), respectively, in the studies whose participants were recruited between the years of 2001 and 2011 and in the studies whose participants were recruited between the years of 2012 and 2021. Significant differences were found in the tests for subgroup differences (*p*=0.002, *I*^2^ = 89.5%). The heterogeneity was increased by the subgroup analysis, which showed that the participants' recruitment period between 2001 and 2011 (*I*^2^ = 99.0% vs 0.0%).

### 5.5. Risk of Bias and Study Quality

The majority of studies (7/8) were rated as having low bias risk. One study was at a moderate risk of bias. The domains on which studies most frequently did poorly were the use of an acceptable case definition in the study (since one was not used in the study) and the use of inappropriate numerators and denominators for the study's parameter of interest (since these concepts were presented, but one or more of them were inappropriate). Each included study's specific quality and bias risk were detailed in Appendix S2. The study's overall results indicated a moderate risk of bias.

### 5.6. Publication Bias

Because there were less than 10 included research studies, we were unable to evaluate the included studies' publication bias. Due to a lack of statistical power, the available tests have a very significant risk of bias when less than 10 studies are included [29].

## 6. Discussion

The World Health Organization's (WHO) global goal of eradicating HIV and viral hepatitis infection by 2030 was put in jeopardy because pregnant women who are triple infected with HIV, HBV, and HCV are at a high risk of passing these viruses from mother to child. This is because people who have three coinfections are more likely to present with lower CD4+ counts, which increases the likelihood that their host immunity will be weakened, which can have more negative effects on pregnancy. According to the results of this systematic review and meta-analysis, the prevalence of triplex coinfections with HIV, HBV, and HCV during pregnancy was 0.03% overall, 0.08% in the community of HIV obstetrics, and 0% in the general obstetric population.

However, the prevalence in the nonobstetric population was lower than in earlier systematic reviews and meta-analyses reported in Africa [30], Iran [31], China [32], and internationally [33, 34]. For instance, Kenfack-Momo et al. reported a pooled prevalence of 0.7% (95% CI = 0.3–1.0) for triplex infections among HIV-infected population in Africa in a systematic study in nonobstetric population [30]. In China, Yu et al. observed a pooled triplex infection prevalence of 3.5% (95% CI 2.4–4.8%) in the nonobstetrics population, with variations detected in terms of age and geographic region [31]. In the nonobstetric group of drug users in Iran, Bagheri Amiri et al. conducted a second comprehensive review and meta-analysis and found a pooled prevalence of 1.25% (95% CI: 0.00–3.01) for triplex infections [32]. In addition, patients who got numerous transfusions and inmates both had a low rate of triplex infections, at 0.01% and 0.28%, respectively, according to the authors of the Bagheri Amiri et al. study [32]. In the nonobstetric population, triplex infection had a pooled global prevalence of 3.00% (95% CI: 1.90–4.73%), according to Chen et al. [33]. In a different global prevalence research in a nonobstetric population, Rashti et al. showed that the prevalence of triplex infections among drug users was 11% (95% CI: 7%–15%) [34].

The decreased prevalence of triplex infection in the current study may be attributable to the study's unique demographic, which of course includes pregnant women. It could also be a result of the passage of time, as more successful methods for reducing mother-to-child transmission of HIV and HBV have been adopted. One of these methods is the elimination of mother-to-child transmission services. The latter could therefore be explained by ongoing efforts to lower mother-to-child transmission and potential HBV vaccine initiatives. Nigeria started its hepatitis B vaccination program in 2004; however, there have not been many program reviews [35, 36].

However, a change in the vaccination policy in Nigeria in February 2015 restricted hepatitis B-birth dose, vaccine administration to only within 24 hours of birth, and Expanded Programme for Immunization (EPI) tools were revised accordingly [34, 36]. Despite this, there is a need to expand national preventative initiatives, which should include educating the public about the risks associated with the spread of triplex infection.

The management of these triplex viruses during pregnancy also depends on the status of HBV and HCV [37]. A diagnosis must be made before treatment can begin because the WHO global hepatitis plan relies on 80% of those with HBV and HCV infection who are eligible for treatment to be treated in order to eradicate viral hepatitis as a public health issue by 2030 [38]. To avoid perinatal transmission of HBV, preventive interventions include identifying pregnant women who are HBV-positive [38]. This is necessary before the advised antiviral medication is given.

In addition, the WHO advises starting highly active antiretroviral therapy (HAART) in HIV patients who are also coinfected with HBV or HCV regardless of their CD4 T lymphocyte count; however, the regimen to use depends on which of these viral infections is present because failing to do so could put the patient at higher risk for hepatotoxicity [5]. Improving HBV vaccine programs, preventing mother-to-child HBV transmission, risk reduction strategies, and optimizing HIV and hepatitis diagnosis and treatment are some of the measures and aims to achieve these goals [39].

In addition to the participant subgroup analysis based on HIV positivity status, the recruiting period-based subgroup analysis is also more important. This is due to the fact that participants were recruited for four of the eight research studies between 2006 and 2011. Given that all three of the documented cases of triplex infection occurred during recruitment periods that began before 2012, this obviously has an impact on the findings of the analysis and the prevalence. This may also be the cause of the recently reported moderate-to-low rates of mother-to-child transmission of HIV and HBV in Nigeria [40].

Following a thorough evaluation of the studies' quality that were a part of this systematic review, we found significant methodological issues with the included research, particularly with regard to the diagnostic criteria, and they were given a “moderate” risk of bias rating. Although the included studies were considered to be of high to moderate quality, their individual study designs and definitions of the outcomes of interest had biases. There was significant heterogeneity (*I*^2^ > 75%) for the main result. A random-effects model was applied to the meta-analysis in order to lessen this effect.

This work has a huge impact on subsequent research. Because none of the included studies had their primary objectives focused on figuring out the prevalence of triplex infections in pregnancy, prospective studies must be created and carried out to assess the prevalence of triplex infections directly and objectively. Only one active study was found [9], and earlier research focused mostly on nonobstetric groups [5, 6, 41]. Therefore, to increase the overall evidence, large cohort studies or carefully planned case-control studies would be suitable study designs.

The seroconversion or seroclearance of triplex infections in Nigeria, however, was not examined in this investigation [9]. The small sample sizes of several of the included studies, which may increase the random error in the pooled prevalence, represent another potential drawback of this analysis. Despite these drawbacks, the current study is the first systematic review and meta-analysis of the available literature that examines the prevalence of triplex infections among pregnant women. To the best of our knowledge, because of this, it serves as the most thorough and recent systematic study of the rate of triplex infections in pregnancy.

## 7. Conclusion

The combined prevalence of HIV, HBV, and HCV triplex coinfections during pregnancy was 0.03%, with a frequency that was noticeably greater in the obstetric population that tested positive for HIV and during the recruiting period that took place before 2012. This prevalence nevertheless necessitates screening for these illnesses and, where necessary, treating and/or immunizing the sick or exposed newborns. To completely eradicate these triplex infections in the obstetric community, health policymakers must continue to implement practical measures. To increase effectiveness and build on current successes in the eradication of triplex infection, it is necessary to increase political will and support, expand treatments based on scientific evidence, and make better use of financing sources.

It is also necessary to do larger, more meticulous prospective investigations using PCR technology for diagnosis. Due to the reported burden of triplex infection among the HIV-positive obstetric population in our meta-analysis, we also advise that antenatal viral screening tests be prioritized to cover HIV, HBV, and HCV infections.

## Figures and Tables

**Table 1:** The characteristics of the included studies.

Rows	Author (references)	Period of participants' recruitment	Region of recruitment	City/area	Study design	Population type	Mean or median age	No. of participants with triplex infection	Sample size	Prevalence (%)
1	Adesina et al. 2010 [16]	Jan 2006 to December 2007	South-West	Ibadan	Retrospective cross sectional	HIV infected	28.86 ± 5.85 years	1	721	0.14
2	Ezechi et al. 2014 [17]	January 2006 to December 2011	South-West	Lagos	Cross sectional	HIV infected	29.5 ± 4.4 years	2	2391	0.08
3	Ikeako et al. 2014 [18]	May 2006 to April 2008	South-East	Enugu	Retrospective cross sectional	General obstetrics	30.1 ± 2.1 years	0	1239	0.0
4	Oga et al. 2018 [19]	February 2015-January 2016	North-Central	Jos	Cohort	HIV infected	Not stated	0	150	0.0
5	Okeke et al. 2012 [20]	January 2007 to December 2009	South-East	Enugu	Retrospective cross-sectional	HIV infected	Not stated	1	401	0.3
6	Omatola et al. 2019 [21]	September 2017 to November 2017	North-Central	Anyigba	Cross sectional	HIV infected	Not stated	0	200	0.0
7	Opaleye et al. 2016 [22]	Not stated, but assumed to be after 2011 since the accepted author version of the manuscript was posted online on 16 April 2015	South-West	Oshogbo	Cross sectional	General obstetrics	Not stated	0	182	0.0
8	Ya'aba et al. 2019 [23]	January 2015 to December 2018	North-Central	Abuja	Cross sectional	General obstetrics	Not stated	0	330	0.0

HIV = human immunodeficiency virus.

**Table 2:** Characteristics of excluded studies.

Study ID	Reasons for exclusion
Balogun et al. 2012 [24]	The study population was not pregnant women with triplex infection but consisted of adult 102 (32 males and 70 females) Nigerian HIV infected patients attending the antiretroviral therapy clinics

Nnakenyi et al. 2020 [5]	The study population was not pregnant women but consisted of 4663 (3024 women and 1639 men) adult patients (aged ≥18 years) with confirmed HIV seropositivity by double ELISA and western blot, who underwent serology testing for both HBsAg and anti-HCV as part of their baseline tests, at the University of Nigeria Teaching Hospital, Nigeria

Forbi et al. 2007 [6]	The study population was not pregnant women with triplex infection but consisted of a cohort of people (83 males and 97 females) living with HIV/AIDS in North-Central Nigeria

Hamza et al. 2013 [25]	The study population was not pregnant women but consisted of four-hundred and forty (178 males and 262 females) consecutive HIV-positive individuals seen at the adult HIV clinic in Aminu Kano Teaching Hospital (AKTH), Kano, North-Western Nigeria

Ogwu-Richard et al. 2015 [26]	The study population was not pregnant but consisted of 183 (100 females and 83 males) HIV-positive persons 15 years of age and above

Otegbayo et al. 2008 [27]	The study population was not pregnant but on eligible HIV-positive treatment-naive patients who presented between August 2004 and February 2007 to the University College Hospital (UCH), Ibadan, Nigeria

Tremeau-Bravard et al. 2012 [28]	The study population was not pregnant women but consisted of 443 (244 women and 199 men) antiretroviral naïve HIV-positive individuals seen at our Gede Foundation clinic in Abuja, Nigeria for HIV/AIDS related infection

**Table 3:** The diagnostic methods, eligibility criteria and risk of bias/quality of the included studies.

Row	Author (references)	Diagnostic method	Inclusion criteria	Exclusion criteria	Quality	Risk of bias
1	Adesina et al. 2010 [16]	ELISA	Pregnant women enrolled into PMTCT	Unavailable result for HBV or HCV	3	Low risk
2	Ezechi et al. 2014 [17]	HIV-WESTERN BLOT; HBV-seropositivity to HBSAG; HCV-antibody positivity to HCV	Pregnant women enrolled into PMTCT	Refusal to give consent	3	Low risk
3	Ikeako et al. 2014 [18]	ELISA	Pregnant women with complete sociodemographic variable, HIV, HBV, and HCV screening results	Women whose mothers were HBV or HCV carriers	2	Low risk
4	Oga et al. 2018 [19]	HBsAg (rapid test), HCV (ELISA)	Pregnant women enrolled into PMTCT	Refusal to give consent	2	Low risk
5	Okeke et al. 2012 [20]	HCV- ELISA + PCR; HBV-ELISA; HIV-not stated	Pregnant women enrolled into PMTCT	Refusal to give consent	4	Moderate risk
6	Omatola et al. 2019 [21]	HCV (ELISA), HBsAg (immunochromatography)	Pregnant women that gave consent	Refusal to give consent	2	Low risk
7	Opaleye et al. 2016 [22]	ELISA, Immunochromatography	All antenatal women	Refusal to give consent	3	Low risk
8	Ya'aba et al. 2019 [23]	Rapid test	Pregnant women enrolled into PMTCT	Not stated	3	Low risk

HIV :  human immunodeficiency virus; ELISA : enzyme linked immuno assay; RDT : rapid diagnostic test.

## Data Availability

All datasets generated and analysed, including the study protocol, search strategy, list of included and excluded studies, data extracted, analysis plans, and quality assessment, are available in the article and upon request from the corresponding author.

## Abbreviations

HAART: Highly active antiretroviral therapy
HBV: Hepatitis B virus
HCV: Hepatitis C virus
HIV: Human immunodeficiency virus
MTCT: Mother-to-child transmission
PCR: Polymerase chain reaction
PRISMA: Preferred reporting items for systematic reviews and meta-analyses
WHO: World Health Organization.

## Supplementary Materials

Supplementary Materials Appendix S1: PubMed search strategy. Appendix S2: risk of bias items and assessment of quality in the included studies.
