# Supplementary material for: A Systematic Review and Meta-Analysis of the Prevalence of Triplex Infections (Combined Human Immunodeficiency Virus, Hepatitis B Virus, and Hepatitis C Virus) among Pregnant Women in Nigeria
Source: Obstet Gynecol Int. 2023 Jul 17;2023:3551297. doi: 10.1155/2023/3551297 (PMC10365920; doi:10.1155/2023/3551297)
Supplement: Supplementary Materials — Appendix S1: PubMed search strategy. Appendix S2: risk of bias items and assessment of quality in the included studies. [file 3551297.f1.docx]

**APPENDIX S1: PUBMED SEARCH STRATEGY**

1. Hepatitis B [mh] OR Hepatitis B virus [mh] OR Hepatitis B Surface Antigens [mh] OR Hepatitis B Antigens [mh] OR Hepatitis B Antibodies [mh] OR Hepatitis B [tiab] OR HBsAg [tiab] OR “Hepatitis Be” [tiab] OR “Be Antigens” [tiab] OR “e Antigens” [tiab] OR “Antigens, e” [tiab] OR HBeAg [tiab] OR HBe Ag-1 [tiab] OR HBe Ag-2 [tiab] OR HBV [tiab] OR “Hep B” [tiab] OR “hbs ag” [tiab] OR “Australia Antigen” [tiab] OR “Antigen, Australia” [tiab]
2. Hepatitis C [mh] OR Hepacivirus [mh: noexp] OR Hepatitis C Antibodies [mh] OR Hepatitis C Antigens [mh] OR Hepatitis C [tiab] OR HCV [ tiab] OR hepaciviru* [tiab] OR “Hep c” [tiab] OR Parenterally Transmitted Non A, Non B Hepatitis [tiab] OR PT-NANBH [tiab] OR Hepatitis Non A, Non B Antigen [tiab]
3. HIV [mh] OR HIV infections [mh] OR HIV [tiab] OR AIDS [tiab] OR “Acquired Immune Deficiency Syndrome” [tiab] OR “Acquired Immunodeficiency Syndrome” [tiab] OR Human Immunodeficiency Virus* [tiab] OR Human T Cell Lymphotropic Virus Type III [tiab] OR Human T Cell Leukemia Virus Type III [tiab] OR Lymphadenopathy Associated Virus* [tiab] OR HTLV-III [tiab] OR T Lymphotropic Virus Type III Infections, Human [tiab] OR Human T Lymphotropic Virus Type III [tiab]
4. Pregnancy [mh] OR Pregnant women [mh] OR Pregnancy Complications [mh] OR Pregnancy Complications, Infectious [mh] OR Pregnan* [tiab] OR Gestation* [tiab]
5. (“Mother-to-Child” [tiab] OR “mother-to-infant” [tiab]) AND transmission* [tiab]
6. (Maternal-fetal [tiab] OR Fetomaternal [tiab] OR Vertical [tiab]) AND (transmission* [tiab] OR infection* [tiab])
7. “Perinatal transmission” [tiab] OR “Maternal-child transmission” [tiab] OR MTCT [tiab] OR “vertical infectious disease transmission” [tiab] OR “Intrauterine transmission” [tiab] OR “utero transmission” [tiab] OR vertical pathogen transmission [tiab]
8. Infectious Disease Transmission, Vertical [mh]
9. #4 OR #5 OR #6 OR #7
10. Nigeria [mh] OR Nigeria* [tiab]
11. #1 AND #2 AND #9 AND #10
12. #1 AND #3 AND #9 AND #10
13. #2 AND #3 AND #9 AND #10
14. #1 AND #2 AND #3 AND #9 AND #10
15. #11 OR #12 OR #13 OR #14
16. Animals [mh] NOT Humans [mesh: noexp]
17. #15 NOT #16
18. "2001/02/01"[PDAT]: "2021/01/31"[PDAT]
19. #17 AND #18

**GOOGLE SCHOLAR SEARCH STRATEGY**

“hepatitis B”|HBV|HBsAg|“hbs ag”|“Hep B” “hepatitis C”|Hepacivirus|“Hep c”|HCV|hepaciviru* HIV|AIDS|“Human Immunodeficiency Virus”|“Acquired Immune Deficiency Syndrome”|“Acquired Immunodeficiency Syndrome” Pregnancy Nigeria.

**Appendix S2: Risk of bias items and assessment of quality in the included studies**

|  |  | **Points Scored** | **Adesina** | **Ezechi** | **Ikeako** | **Oga** | **Okeke *et al.*** | **Omatola** | **Opalaye** | **Ya’aba** |
| --- | --- | --- | --- | --- | --- | --- | --- | --- | --- | --- |
| **1. Was the study’s target population a close representation of the national population in relation to relevant variables, e.g. age, sex, occupation?** | Yes (LOW RISK): The study’s target population was a close representation of the national population. | 0 | 0 | 0 | 0 | 0 | 0 | 0 | 0 | 0 |
|  | No (HIGH RISK): The study’s target population was clearly NOT representative of the national population. | 1 |  |  |  |  |  |  |  |  |
| **2. Was the sampling frame a true or close representation of the target population?** | Yes (LOW RISK): The sampling frame was a true or close representation of the target population. | 0 | 0 | 0 | 0 | 0 | 0 | 0 | 0 | 0 |
|  | No (HIGH RISK): The sampling frame was NOT a true or close representation of the target population. | 1 |  |  |  |  |  |  |  |  |
| **3. Was some form of random selection used to select the sample, OR, was a census undertaken?** | Yes (LOW RISK): A census was undertaken, OR, some form of random selection was used to select the sample (e.g. simple random sampling, stratified random sampling, cluster sampling, systematic sampling). | 0 | 0 | 1 | 0 | 0 | 1 | 0 |  | 0 |
|  | No (HIGH RISK): A census was NOT undertaken, AND some form of random selection was NOT used to select the sample. | 1 |  | 1 |  |  |  |  | 1 |  |
| **4. Was the likelihood of non-response bias minimal?** | Yes (LOW RISK): The response rate for the study was ≥75%, OR, an analysis was performed that showed no significant difference in relevant demographic characteristics between responders and non- responders | 0 | 0 | 0 | 0 | 0 | 0 |  | 0 | 0 |
|  | No (HIGH RISK): The response rate was <75%, and if any analysis comparing responders and non-responders was done, it showed a significant difference in relevant demographic characteristics between responders and non-responders | 1 |  |  |  |  |  | 1 |  |  |
| **5. Were data collected directly from the subjects (as opposed to a proxy)?** | Yes (LOW RISK): All data were collected directly from the subjects. | 0 | 0 | 0 | 0 | 0 |  | 0 |  | 0 |
|  | No (HIGH RISK): In some instances, data were collected from a proxy | 1 |  |  |  |  |  |  | 1 |  |
| **6. Was an acceptable case definition used in the study?** | Yes (LOW RISK): An acceptable case definition was used. | 0 |  |  |  |  |  |  |  |  |
|  | No (HIGH RISK): An acceptable case definition was NOT used | 1 | 1 | 1 | 1 | 1 |  | 1 | 1 | 1 |
| **7. Was the study instrument that measured the parameter of interest (e.g. prevalence of low back pain) shown to have reliability and validity (if necessary)?** | Yes (LOW RISK): The study instrument had been shown to have reliability and validity (if this was necessary), e.g. test-re- test, piloting, validation in a previous study, etc. | 0 | 0 | 0 | 0 | 0 | 0 | 0 | 0 | 0 |
|  | No (HIGH RISK): The study instrument had NOT been shown to have reliability or validity (if this was necessary). | 1 |  |  |  |  |  |  |  |  |
| **8. Was the same mode of data collection used for all subjects?** | Yes (LOW RISK): The same mode of data collection was used for all subjects. | 0 | 0 | 0 | 0 | 0 | 0 | 0 | 0 | 0 |
|  | No (HIGH RISK): The same mode of data collection was NOT used for all subjects. | 1 |  |  |  |  |  |  |  |  |
| **9. Were the numerator(s) and denominator(s) for the parameter of interest appropriate** | Yes (LOW RISK): The paper presented appropriate numerator(s) AND denominator(s) for the parameter of interest (e.g. the prevalence of low back pain). | 0 |  |  |  |  |  |  |  |  |
|  | No (HIGH RISK): The paper did present numerator(s) AND denominator(s) for the parameter of interest but one or more of these were inappropriate. | 1 | 1 | 1 | 1 | 1 | 1 | 1 | 1 | 1 |
| **10. Summary on the overall risk of study bias** | Low Risk (0 to 3) | TOTAL | **3** | **3** | **2** | **2** | **5** | **2** | **3** | **3** |
|  | Moderate Risk (4 to 6) |  |  |  |  |  |  |  |  |  |
|  | High Risk (7 to 9) |  |  |  |  |  |  |  |  |  |
